# Individualized estimation of arterial carbon dioxide partial pressure using machine learning in children receiving mechanical ventilation

**DOI:** 10.1186/s12887-024-04642-0

**Published:** 2024-02-29

**Authors:** Hye-Ji Han, Bongjin Lee, June Dong Park

**Affiliations:** 1 Department of Pediatrics, Seoul National University College of Medicine, Seoul National University Children’s Hospital, Seoul, 03080 Republic of Korea; 2 Innovative Medical Technology Research Institute, Seoul National University Hospital, Seoul, Republic of Korea

**Keywords:** Machine learning, Blood gas analysis, Capnography, Mechanical ventilation, Respiratory Dead Space

## Abstract

**Background:**

Measuring arterial partial pressure of carbon dioxide (PaCO_2_) is crucial for proper mechanical ventilation, but the current sampling method is invasive. End-tidal carbon dioxide (EtCO_2_) has been used as a surrogate, which can be measured non-invasively, but its limited accuracy is due to ventilation-perfusion mismatch. This study aimed to develop a non-invasive PaCO_2_ estimation model using machine learning.

**Methods:**

This retrospective observational study included pediatric patients (< 18 years) admitted to the pediatric intensive care unit of a tertiary children’s hospital and received mechanical ventilation between January 2021 and June 2022. Clinical information, including mechanical ventilation parameters and laboratory test results, was used for machine learning. Linear regression, multilayer perceptron, and extreme gradient boosting were implemented. The dataset was divided into 7:3 ratios for training and testing. Model performance was assessed using the R^2^ value.

**Results:**

We analyzed total 2,427 measurements from 32 patients. The median (interquartile range) age was 16 (12−19.5) months, and 74.1% were female. The PaCO2 and EtCO2 were 63 (50−83) mmHg and 43 (35−54) mmHg, respectively. A significant discrepancy of 19 (12–31) mmHg existed between EtCO_2_ and the measured PaCO_2_. The R^2^ coefficient of determination for the developed models was 0.799 for the linear regression model, 0.851 for the multilayer perceptron model, and 0.877 for the extreme gradient boosting model. The correlations with PaCO_2_ were higher in all three models compared to EtCO_2_.

**Conclusions:**

We developed machine learning models to non-invasively estimate PaCO_2_ in pediatric patients receiving mechanical ventilation, demonstrating acceptable performance. Further research is needed to improve reliability and external validation.

## Background

Evaluating adequate oxygen and carbon dioxide (CO_2_) exchange is crucial for managing patients receiving mechanical ventilation. The arterial partial pressure of CO_2_ (PaCO_2_) is commonly used for evaluation, but its invasive measurement via arterial blood sampling can be painful and cause iatrogenic complications such as mechanical damage of arteries, anemia, embolism, thrombosis, or nerve injury. In general, as the difficulty of the procedure increases in smaller infant, the degree of complications may become more severe [1]. Therefore, this highlights the need for a non-invasive method to estimate PaCO_2_ in children.

End-tidal CO_2_ (EtCO_2_) and transcutaneous CO_2_ (TCO_2_) monitoring are feasible alternatives. EtCO_2_ reflects exhaled CO_2_, but ventilation-perfusion mismatch limits its accuracy as a surrogate for PaCO_2_. Factors like dead-space ventilation, severe atelectasis, and intrapulmonary shunts can influence its reliability in various clinical settings. Therefore, bedside practice often utilizes the EtCO_2_ trend to estimate PaCO_2_ trends rather than relying on a single value [2–4]. TCO_2_, measuring the partial pressure of CO_2_ in arteriolarized capillaries through skin warming, also has limitations that make it difficult to use it widely in clinical practice, especially in acute medical settings [5, 6].

Few studies have attempted to estimate the dead space fraction using the difference between EtCO_2_ and PaCO_2_, which was even described as an independent risk factor associated with mortality [7, 8]. However, developing a robust formula for this purpose remains challenging due to the influence of various physiological factors [7–12]. Recent research has demonstrated the successful application of artificial intelligence for individualized estimation and prediction of complex factors, overcoming limitations of conventional methods [13–15].

This study aimed to develop machine learning models based on non-invasively collected data and compare their performance to estimate PaCO_2_ more accurately in mechanically ventilated children.

## Methods

### Study setting and data collection

This retrospective cross-sectional observational study was conducted in the pediatric intensive care unit (PICU) of a university-affiliated children’s hospital. Patients aged less than 18 years who were admitted to the PICU and received mechanical ventilation between January 2021 and June 2022 were eligible for inclusion. Patients who underwent extracorporeal membrane oxygenation, which allows oxygenation and CO_2_ elimination using methods other than mechanical ventilation, were excluded. Additionally, patients who did not have PaCO_2_ measured during mechanical ventilation were excluded.

Data used in this study were retrieved from our institution’s data warehouse. Demographic information, clinical characteristics (systolic blood pressure [BP], diastolic BP, heart rate [HR], respiratory rate [RR], peripheral oxygen saturation [SpO_2_], height, weight, and EtCO_2_), and ventilator parameters (minute ventilation, peak inspiratory pressure, fraction of inspired oxygen [FiO_2_], positive end-expiratory pressure [PEEP], and mean airway pressure [MAP]) were collected. Blood gas analysis results, representing the only invasive data in this study, were collected for training the machine learning model.

This study was conducted in accordance with the principles of the Declaration of Helsinki. The protocol of this study was reviewed by the Institutional Review Board of Seoul National University Hospital. This study was recognized as a minimal risk study by the above committee because it used only pseudonymous information and did not collect personally identifiable information. Therefore, for the above reasons, the above committee waived the need for written informed consent and approved the conduct of this study (approval no. H-2307-160-1452).

### Data preprocessing

Among the collected data, cases containing duplicates or missing values were excluded. A limitation of retrospectively collected blood gas analysis results is the inability to definitively differentiate between arterial and venous blood samples. Therefore, we assumed arterial blood when the arterial partial pressure of oxygen (PaO_2_) was equal to or greater than 40 mmHg, thereby including cases with hypoxemia. Additionally, measurements outside the physiological range, suggesting errors in recording or measurement, were excluded. These criteria included: HR < 30 beats/minute or > 300 beats/minute, RR < 5 breaths/minute or > 120 breaths/minute, EtCO_2_ < 20 mmHg, and FiO_2_ < 21%. For clinical information and ventilator parameters, time values were converted to hours by discarding minutes and seconds. Laboratory findings were integrated into a one-hour window. To improve model performance, FiO_2_, which exhibits a known non-linear relationship with PaCO_2_, was log-transformed. Patients’ BPs were converted to percentiles based on age, sex, and height [16]. Pulse pressure and oxygenation saturation index (OSI) were calculated using the collected variables following the method outlined [17]. Data preprocessing was performed using R statistical packages R 4.3.1.

### Strategy of analysis

The primary outcome of this study was the estimation performance of the developed models. Performance was compared with the R^2^ values and visualized through the Bland-Altman analysis with 95% limits of agreement and scatter plots. The measured PaCO_2_ was referred to as the gold standard, and the machine learning-derived estimated CO_2_ was served as the comparator. The correlation coefficients between EtCO_2_ and the measured PaCO_2_ were presented with *P*-values as a secondary outcome.

Demographics and characteristics were presented as median (interquartile range) and compared using the Wilcoxon signed-rank test for non-normally distributed categorical variables and the chi-square test for other categorical variables. To account for the discrepancy between PaCO_2_ and EtCO_2_, the absolute values of PaCO_2_-EtCO_2_ were calculated. Correlations among the values were evaluated using Spearman’s correlation coefficient. Linear regression analysis of PaCO_2_ and EtCO_2_ was performed to determine R^2^ values. The normality of residuals was assessed using the Shapiro-Wilk test before conducting the Bland-Altman analysis. Statistical analyses were performed using R software version 4.3.1., *P* -values less than 0.05 were considered statistically significant, and a confidence level of 95% was chosen for analysis.

### Model development and validation

Using collinearity analysis of the classical linear regression and feature importance analysis in the models, the following variables that can be non-invasively retrievable in clinical settings were selected for machine learning: weight, percentile of systolic and diastolic BP, RR, HR, EtCO_2_, minute ventilation, log-transformed FiO_2_, SpO_2_, MAP and PEEP. To minimize the outliers’ impact, the variables were scaled according to the quantile range.

To mitigate potential overfitting and selection bias from the varying numbers of individual measurements per patient, each patient’s data was split into a 7:3 ratio for training and validation. Machine-learning models were developed using three algorithms: linear regression, multi-layer perceptron (MLP), and extreme gradient boosting (XGB). The final model of multivariate linear regression was built based on the Akaike information criterion, and multicolinearity was assessed using the variance inflation factor. Optimal hyperparameters for MLP and XGB models were extracted through a grid search with five-fold cross-validation [18, 19]. The MLP model was designed with two hidden layers: 50 nodes in the first and 10 in the second. ReLU activation and Adam solver were used. The XGB model had a maximum decision tree depth of seven, a learning rate of 0.01, and 500 estimators. Feature importance in the XGB model was determined using Shapley additive explanations (SHAP) values, reflecting the impact of various features on the model [20]. Machine learning was implemented using Python version 3.10.4 (Python Software Foundation, Beaverton, OR, USA; https://www.python.org) and open libraries such as Pandas, Keras, Pytorch, Numpy and Scikit-learn.

## Results

### Baseline characteristics

**Fig. 1:**
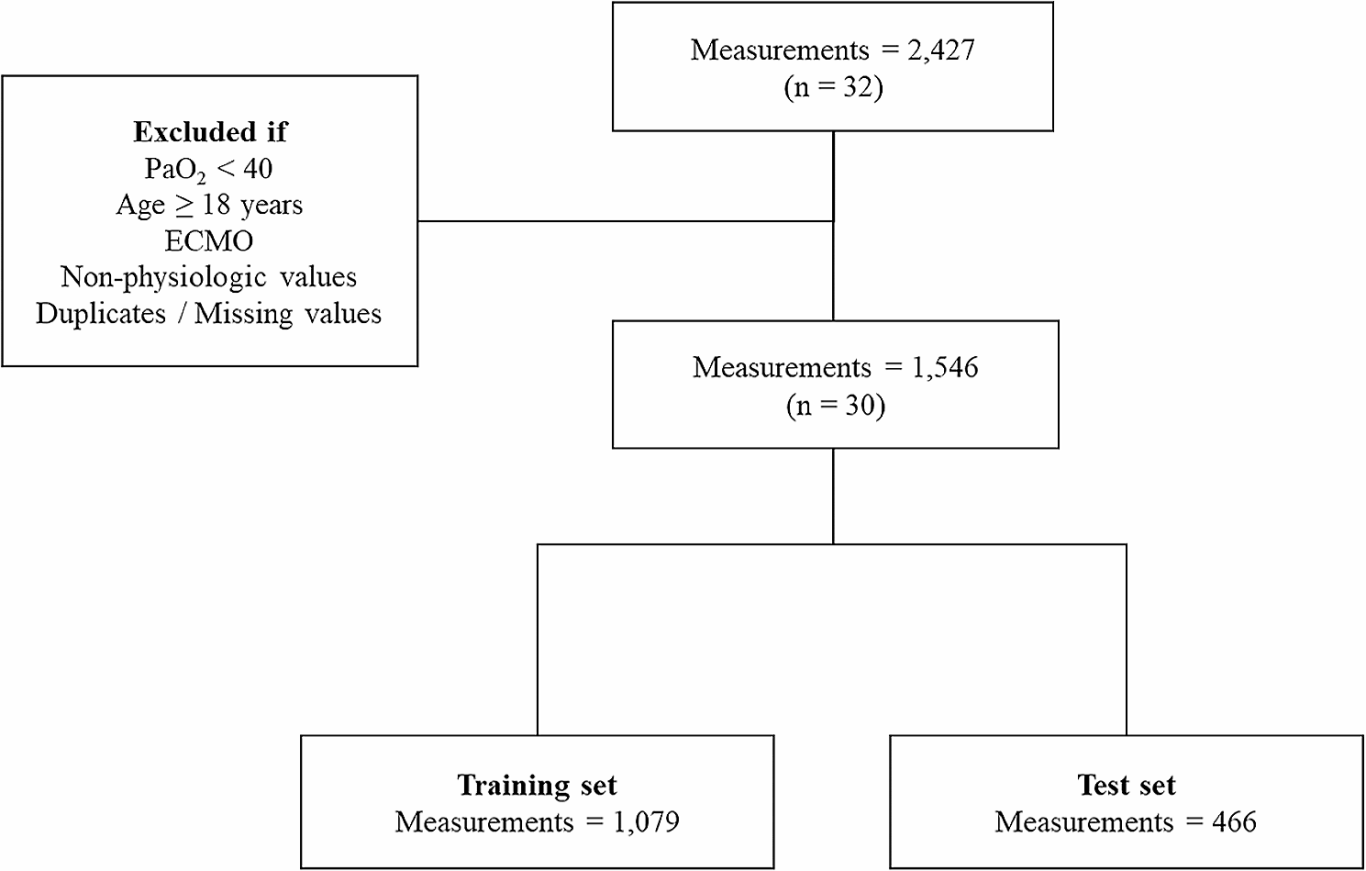
Flowchart of the study cohort used for model development and validation. PaO_2_: arterial partial pressure of oxygen; ECMO: extracorporeal membrane oxygenation

From 32 pediatric patients, we collected a total of 2,427 measurements. After applying the exclusion criteria, 1,546 measurements from 30 patients remained for analysis (Fig. 1). Patient demographics and baseline characteristics were presented in Table 1.

**Table 1:** Demographics and characteristics of patients

Variables	Entire dataset	Training set	Test set	*P*-value
	(*n* = 1,546)	(*n* = 1,079)	(*n* = 466)	
Age, months	16 (12−19.5)	16 (12−18)	16 (12−20)	0.880
Female	1,145 (74.1)	799 (74.1)	345 (74.0)	0.995
Percentile of systolic BP	0.5 (0.1−0.9)	0.5 (0.1−0.9)	0.5 (0.2−0.9)	0.650
Percentile of diastolic BP	0.8 (0.5−1.0)	0.8 (0.6−1.0)	0.8 (0.5−1.0)	0.490
Pulse pressure, mmHg	33 (26−42)	33 (26−41)	34 (26−43)	0.144
Respiratory rate, breaths/minute	25 (17−32)	25 (17−32)	25 (17−32)	0.635
Heart rate, beats/minute	110 (97−129)	110 (96−129)	110 (97−129)	0.725
EtCO_2_, mmHg	43 (35−54)	42 (34−54)	43 (35−54)	0.645
SpO_2_, %	96 (90−100)	96 (90−100)	96 (90−100)	0.989
Minute ventilation, L/minute	1.4 (1.0−2.2)	1.4 (1.0−2.2)	1.4 (1.0−2.2)	0.899
FiO_2_, %	50 (36.3−69.2)	50.1 (38.3−69.3)	49.4 (34.8−69.1)	0.409
MAP, cmH_2_O	14.1 (11.5−16.9)	14.0 (11.6−16.9)	14.2 (11.5−16.7)	0.752
PIP, cmH_2_O	26.8 (21.8−32.6)	26.7 (21.8−32.7)	27.0 (21.8−32.5)	0.992
PEEP, cmH_2_O	10 (8−12)	10 (8−12)	10 (8−12)	0.808
PaCO_2_, mmHg	63 (50−83)	63 (50−83)	63 (50−83)	0.889
PaO_2_/FiO_2_, mmHg	119.4 (82.6−202.2)	118.6 (82.4−198.8)	120.2 (83.1−217.4)	0.586
(PaCO_2_ - EtCO_2_), mmHg	19 (12−31)	19 (12−31)	20 (12−30)	0.678
Oxygenation saturation index	7.2 (4.4−11.8)	7.2 (4.5−11.8)	7.1 (4.2−11.5)	0.488

Continuous variables are expressed as medians (interquartile ranges), and categorical variables are expressed as numbers (percentages). BP, blood pressure; EtCO_2_, end-tidal carbon dioxide; SpO_2_, peripheral oxygen saturation; FiO_2_, fraction of inspired oxygen; MAP, mean airway pressure; PIP, peak inspiratory pressure; PEEP, positive end-expiratory pressure; PaCO_2_, arterial partial pressure of carbon dioxide; PaO_2_, arterial partial pressure of oxygen; (PaCO_2_ - EtCO_2_), discrepancy between PaCO_2_ and EtCO_2_.

The age at admission was 16 (12−19.5) months and female predominance was observed. The OSI indicated mild to moderate acute respiratory distress syndrome (ARDS), and the proportion of patients with OSI ≥ 12, corresponding to severe ARDS, was 31.46% [17]. The percentiles of systolic and diastolic BP suggested that most patients were not hypotensive and had BP above the fifth percentile. The majority of patients were hypercapnic and the discrepancy between PaCO_2_ and EtCO_2_ was 19 (12−31) mmHg. The PaO_2_/FiO_2_ ratio was 119.4 (82.6−202.2) mmHg.

### Main outcomes

**Fig. 2:**
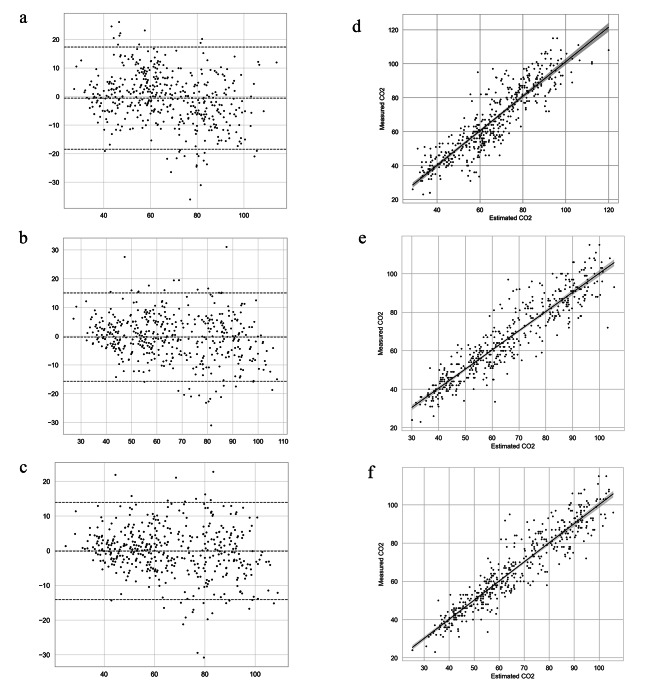
Performance and bias of the machine learning models. The Bland-Altman analysis for the linear regression model, the multi-layer perceptron (MLP) model, and the extreme gradient boosting (XGB) model are shown in (**A**), (**B**), and (**C**), respectively. The scatter plots for the linear regression model, the MLP model, and the XGB model are exhibited in (**D**), (**E**), and (**F**), respectively. CO2: carbon dioxide

The R^2^ values and correlation coefficients for the linear, MLP, and XGB models were summarized in Table 2. Bland-Altman analyses were performed for the models using scaled data. The linear regression model exhibited a mean difference of -0.574 with a 95% limit of agreement (LOA) ranging from − 18.449 to 17.301. The MLP model improved upon this, showing a mean difference of -0.108 with a tighter 95% LOA of -14.107 to 13.892. The XGB model achieved the best performance, with a mean difference of -0.340 and a narrow 95% LOA of -15.716 to 15.036. This is further confirmed by the scatter plot, which demonstrates minimal bias (Fig. 2).

**Table 2:** The correlation between the estimated CO_2_ and the measured PaCO_2_

	Linear regression	MLP	XGB
Correlation coefficient	0.94 (*P* < 0.001)	0.93 (*P* < 0.001)	0.94 (*P* < 0.001)
R^2^ value	0.799	0.851	0.877

Correlations were estimated using the Spearman correlation coefficient, and R^2^ values were calculated to compare the developed models. MLP, multi-layer perceptron; XGB, extreme gradient boosting.

**Fig. 3:**
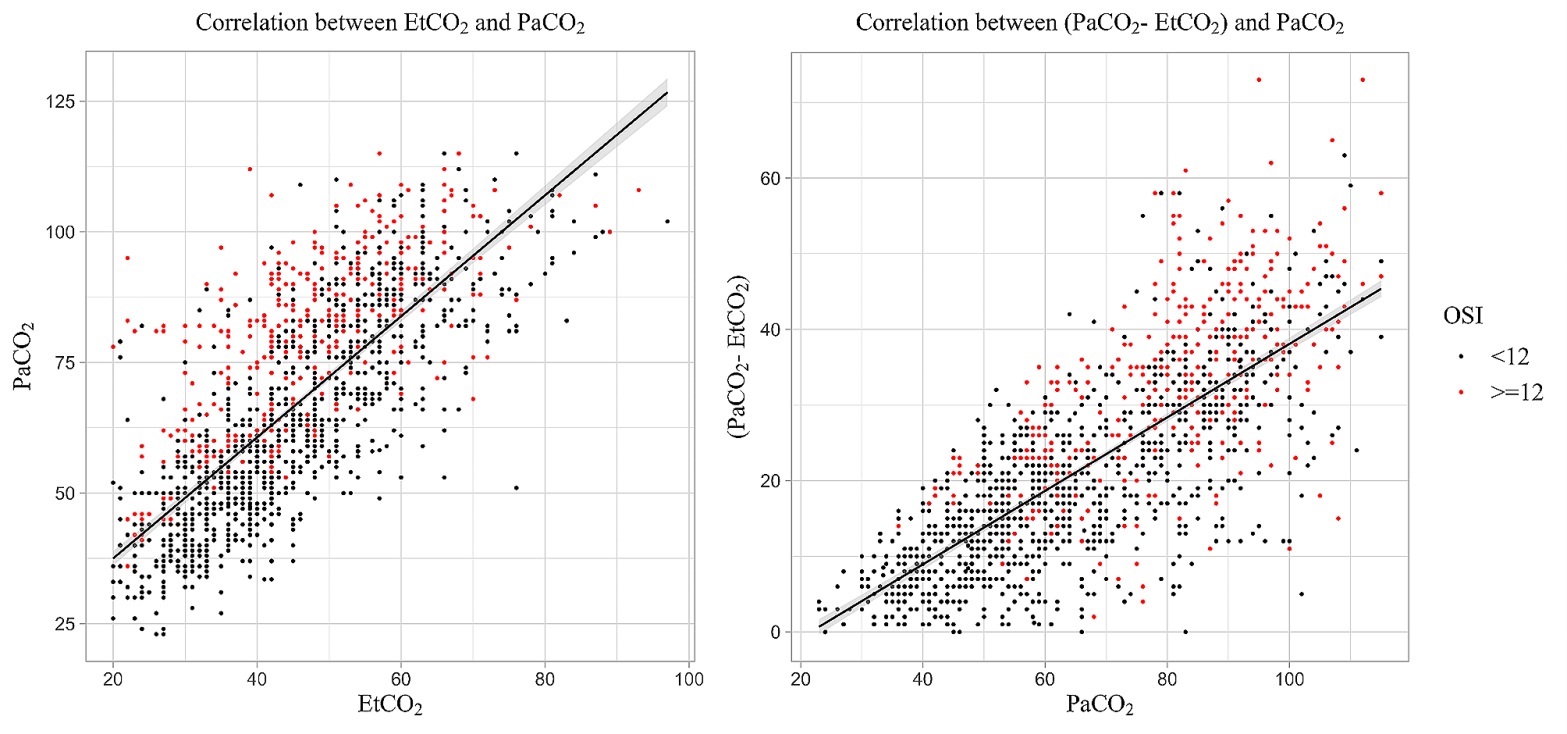
Scatter plot showing the correlation of PaCO_2_ and EtCO_2_. The Spearman correlation coefficient was 0.78, and the R^2^ value was 0.58. The plot reveals a poor correlation between PaCO2 and EtCO_2_ as PaCO_2_ increases. The values of those who belong to severe acute respiratory distress syndrome, defined by the oxygenation saturation index ≥ 12, were represented with red dots. OSI: oxygenation saturation index; (PaCO_2_ - EtCO_2_): discrepancy between PaCO_2_ and EtCO_2_

**Fig. 4:**
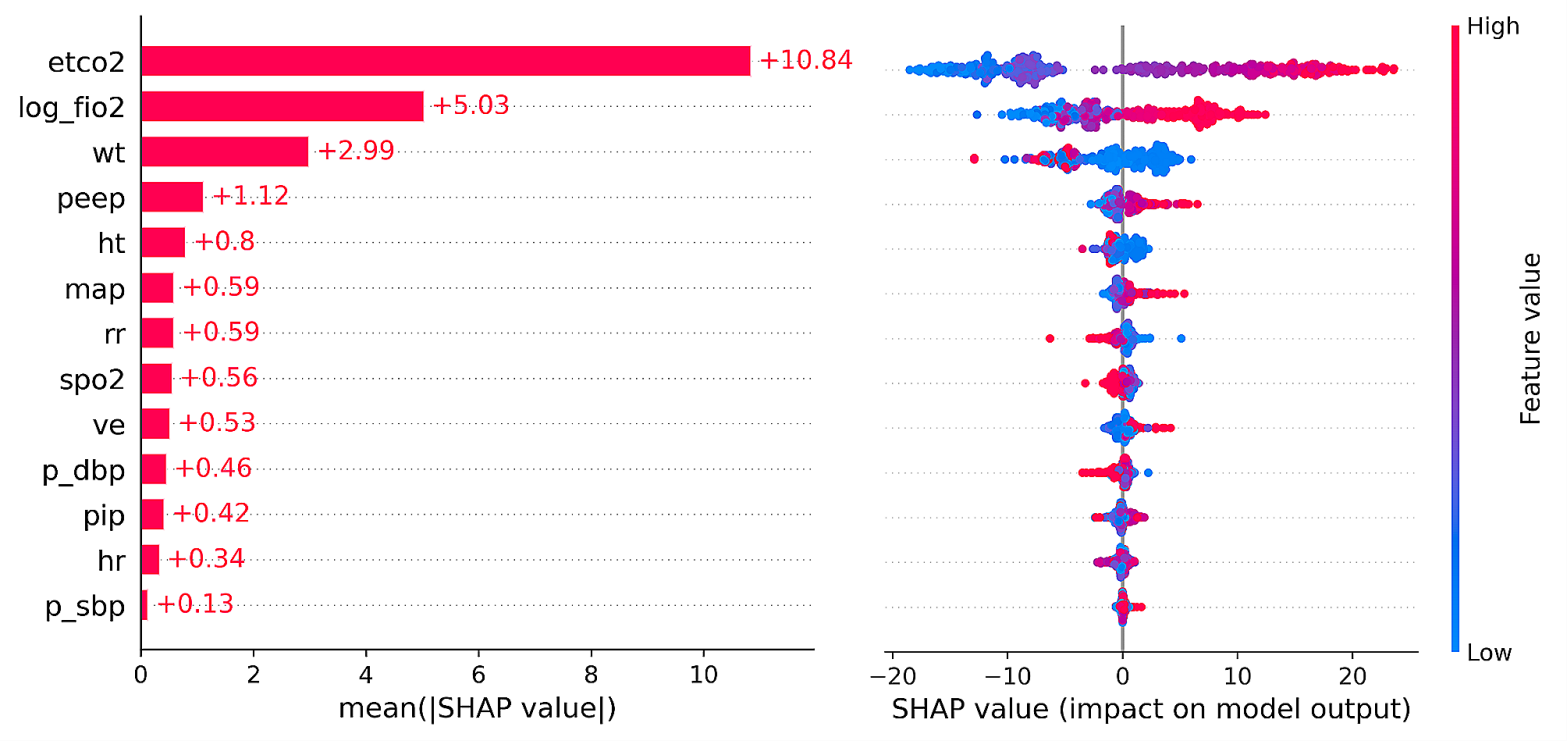
The feature importance in the XGB model. The relative importance of each feature was measured by Shapley additive explanations values. etco2: end-tidal carbon dioxide; log_fio2: log-transformed fraction of inspired oxygen; wt: weight; peep: positive end-expiratory pressure; ht: height; map: mean airway pressure, rr: respiratory rate; spo2: peripheral oxygen saturation; ve: minute ventilation; p_dbp: percentile of diastolic blood pressure; pip: peak inspiratory pressure; hr: heart rate; p_sbp: percentile of systolic blood pressure; |SHAP|: the absolute values of Shapley additive explanations; SHAP: Shapley additive explanations

The Shapiro-Wilk test performed on residuals yielded *P*-values of 0.042, < 0.001, and < 0.001 for the linear regression, MLP, and XGB models, respectively. The developed models presented high correlations with PaCO_2_. In comparison, EtCO_2_ and PaCO_2_ displayed a correlation coefficient of 0.78 (*P* < 0.001) and an R^2^ value of 0.58, highlighting the superior performance of machine learning models. Notably, the discrepancy between EtCO_2_ and PaCO_2_ increased with higher PaCO_2_ values, and the group with OSI ≥ 12 was more prevalent at a greater discrepancy between EtCO_2_ and PaCO_2_ (Fig. 3). The feature importance of the XGB model, which exhibited the highest performance, is visualized in Fig. 4.

## Discussion

This study aimed to develop a non-invasive machine learning model for estimating PaCO_2_ in critically ill children receiving mechanical ventilation. We focused on minimizing the difference between PaCO_2_ and EtCO_2_ through machine learning, recognizing the significant impact that dead space ventilation or perfusion status can have on their correlation [7, 21, 22].

In the study cohort, the correlation coefficient between EtCO_2_ and PaCO_2_ was 0.78 (*P* < 0.001), with an R^2^ value of 0.58. This was in with previous reporting R^2^ values ranging from 0.4 to 0.9, depending on disease entities and ventilator modes [3, 4, 23].

Cross-sectional studies and clinical trials conducted in both adults and pediatric patients have investigated factors influencing the difference between EtCO_2_ and PaCO_2_. These studies suggest a negative relationship with PaO_2_/FiO_2_ [3, 23]. However, these studies primarily included patients with relatively small discrepancies between PaCO_2_ and EtCO_2_, and limited representation of patients with PaO2/FiO2 ≤ 200 mmHg. McDonald et al. [23] observed a discrepancy of 6.8 mmHg with a standard deviation of 6.4 mmHg, while Wang et al. [3] reported an even smaller discrepancy of 1.86 ± 7.42 mmHg in patients undergoing synchronized intermittent mandatory ventilation.

In contrast to these previous studies, our study included patients with a larger discrepancy between PaCO_2_ and EtCO_2_ of 19 (12−31) mmHg and a lower PaO_2_/FiO_2_ of 119.4 (82.6−202.2) mmHg. Even though the median OSI was in accordance with mild to moderate ARDS, the proportion of the group with OSI ≥ 12 was considerable. In our cohort, severely ill children with OSI ≥ 12 were observed more frequently at a higher discrepancy between EtCO_2_ and PaCO_2,_ especially in hypercapnic groups.

Additionally, PaCO_2_ levels in previous studies were reported to be lower than those observed in this study. Razi et al. [4] reported high accuracy (R^2^ > 0.8) for patients with a mean of PaCO_2_ of 45.8 ± 17.1 mmHg in synchronized intermittent mandatory ventilation mode. In our study, the PaCO_2_ was hypercapnic at 63 (50−83) mmHg, with an OSI of 7.2 (4.4−8.6). As respiratory failure and significant dead space are relatively common in critically ill patients, the performance of the models estimating PaCO_2_ may depend on their reliability in hypercapnic and hypoxemic patient groups. Utilizing artificial intelligence, we built more customized estimations compared to the traditional risk stratification methods [13–15]. The XGB model achieved the highest R^2^ value of 0.877 among the three models, all of which demonstrated better correlations with PaCO_2_ than EtCO_2_, even in patients with hypercapnia and hypoxemia.

Nevertheless, several limitations need to be acknowledged. Firstly, utilizing PaO_2_ as a cutoff to identify arterial blood in blood gas analysis might have led to the inclusion of venous blood results and potential exclusion of true arterial blood samples. This limitation is inherent to retrospective studies and arises due to the lack of explicit and clear labeling. Secondly, this study was conducted within a single-institution PICU. Consequently, external validation by other institutions or utilizing different measurement equipment was performed. Thirdly, despite separating training and testing data for each patient, repeated sampling from the individuals might have resulted in overfitting. Further studies with external validation and prospective cohorts are required. At last, given that our models were developed based on data retrieved in a 1-hour window, future analyses based on real-time data would prompt the clinical application of the developed model.

One of the key elements influencing the difference between EtCO_2_ and PaCO_2_ is the heterogeneous distribution of ventilation-perfusion mismatch among alveoli [21]. Including capnographic waveforms as a variable is expected to improve model performance [22]. At last, although interpreting relevant parameters is crucial for understanding gas exchange and dead space ventilation, the “black box” nature of machine learning makes it difficult to access detailed information regarding the developed model’s mechanisms. Regarding this limitation, we tried to incorporate an explanation using SHAP values; however, it remains still challenging to provide an intuitive interpretability.

In conclusion, we successfully developed a machine learning-based model capable of non-invasively estimating PaCO_2_ in critically ill pediatric patients receiving mechanical ventilation. The model demonstrated acceptable performance; however, external validation has not yet been performed, and further improvement in performance through follow-up studies is promising.

## Data Availability

No datasets were generated or analysed during the current study.

## Abbreviations

ARDS: Acute respiratory distress syndrome
BP: Blood pressure
CO_2_: Carbon dioxide
EtCO_2_: End-tidal carbon dioxide
FiO_2_: Fraction of inspired oxygen
HR: Heart rate
LOA: Limit of agreement
MAP: Mean airway pressure
MLP: Multi-layer perceptron
OSI: Oxygenation saturation index
PaCO_2_: Arterial partial pressure of carbon dioxide
PaO_2_: Arterial partial pressure of oxygen
PEEP: Positive end-expiratory pressure
PICU: Pediatric intensive care unit
RR: Respiratory rate
SHAP: Shapley additive explanations values
SpO_2_: Peripheral oxygen saturation
TCO_2_: Transcutaneous carbon dioxide
XGB: Extreme gradient boosting
