# Correction: Individualized estimation of arterial carbon dioxide partial pressure using machine learning in children receiving mechanical ventilation

**DOI:** 10.1186/s12887-024-04772-5

**Published:** 2024-04-25

**Authors:** Hye-Ji Han, Bongjin Lee, June Dong Park

**Affiliations:** 1 Department of Pediatrics, Seoul National University College of Medicine, Seoul National University Children’s Hospital, Seoul, 03080 Republic of Korea; 2 Innovative Medical Technology Research Institute, Seoul National University Hospital, Seoul, Republic of Korea

**Correction:** ***BMC Pediatr*** **24, 149 (2024)**

10.1186/s12887-024-04642-0

Following publication of the original article [1], the authors would like to correct the IRB approval number from “H-2307-160-1452” to “H-2207-180-1344”.
